# Navigating Abdominal Volvulus: A Comprehensive Review of Management Strategies

**DOI:** 10.7759/cureus.57978

**Published:** 2024-04-10

**Authors:** Simran Chauhan, Raju K Shinde, Yashraj Jain

**Affiliations:** 1 General Surgery, Jawaharlal Nehru Medical College, Datta Meghe Institute of Higher Education and Research, Wardha, IND; 2 General Surgery, Rajshree Nursing Home, Ashoknagar, IND

**Keywords:** complications, multidisciplinary approach, diagnostic evaluation, surgical management, gastrointestinal obstruction, abdominal volvulus

## Abstract

Abdominal volvulus represents a critical condition characterized by the abnormal twisting of the GI tract, potentially leading to obstruction and vascular compromise. Prompt recognition and appropriate management are essential to prevent complications and improve patient outcomes. This comprehensive review examines the anatomy, pathophysiology, clinical presentation, and diagnostic evaluation of, and management strategies for abdominal volvulus. Non-operative techniques, including detorsion and decompression, as well as surgical interventions, such as laparoscopic and open approaches, are discussed. Additionally, the importance of multidisciplinary collaboration and postoperative care is emphasized. Despite significant advancements, unresolved issues remain, necessitating further research to refine diagnostic and therapeutic approaches. Future directions, including exploring emerging technologies, offer promise for enhancing the management of this challenging condition. Overall, this review provides clinicians with valuable insights into the optimal management of abdominal volvulus, aiming to improve patient outcomes and enhance clinical practice.

## Introduction and background

Abdominal volvulus refers to the abnormal twisting or torsion of a segment of the GI tract, leading to obstruction and potentially compromised blood flow [1]. This condition typically involves the intestines but can also affect other abdominal organs, such as the stomach or colon. The twisting of the bowel can result in severe complications, including ischemia, necrosis, and perforation if not promptly treated [2]. Understanding the management strategies for abdominal volvulus is crucial due to the potentially life-threatening nature of the condition. Prompt recognition and appropriate intervention are essential to prevent complications and improve patient outcomes. Moreover, effective management requires a multidisciplinary approach involving surgeons, gastroenterologists, radiologists, and critical care specialists to tailor treatment to individual patient needs [3].

This comprehensive review explores the various management strategies available for abdominal volvulus. It will cover the anatomy and pathophysiology underlying the condition, different types of volvulus, clinical presentation, diagnostic evaluation, and non-operative and surgical management approaches. Additionally, the review will discuss postoperative care, potential complications, recent advances, and future directions in the field. Through an in-depth examination of these topics, this review seeks to provide clinicians with valuable insights into the optimal management of abdominal volvulus.

## Review

Classification of abdominal volvulus

Types of Abdominal Volvulus (Based on Anatomical Location)

**Figure 1:**
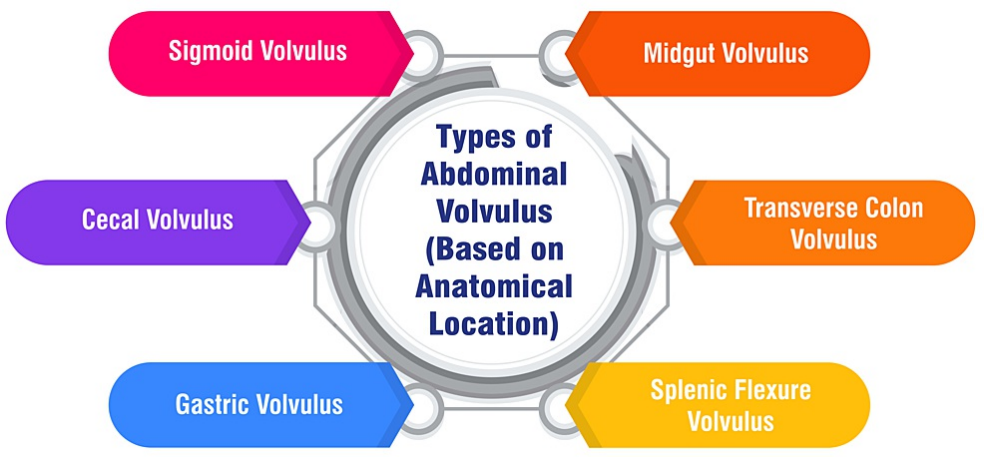
Types of abdominal volvulus (based on anatomical location) The image is created by the corresponding author

Abdominal volvulus manifests in various forms, each with distinct anatomical involvement and clinical presentations. Sigmoid volvulus, the most prevalent form of colonic volvulus, entails a twist in the sigmoid colon [2,4]. Cecal volvulus encompasses a twist of the ileum, cecum, and proximal ascending colon around the mesentery, with variations including an axial twist or folding of the cecum upward toward the hepatic [2,4]. Gastric volvulus arises from the torsion of the stomach around its mesentery, resulting in bowel obstruction and symptoms such as intractable retching, upper abdominal pain, and the inability to pass a nasogastric tube, known as Borchardt's triad [5]. Midgut volvulus primarily affects the pediatric population and involves twisting the small bowel around its mesenteric axis, often associated with malrotation [2]. Transverse colon volvulus, though less common than sigmoid volvulus, presents with symptoms such as abdominal pain and distension caused by a twist in the transverse colon [6]. Splenic flexure volvulus, a rare occurrence, is characterized by the twisting of the splenic flexure of the colon, typically observed in individuals with chronic constipation [7]. These various forms of abdominal volvulus necessitate a tailored approach to diagnosis and management based on their unique anatomical characteristics and clinical implications. Figure 1 shows types of abdominal volvulus (based on anatomical location).

Clinical Implications of Different Types

Sigmoid volvulus, frequently associated with neurological pathologies such as multiple sclerosis and Parkinson's disease [5], manifests with symptoms such as abdominal distension, decreased appetite, and reduced bowel output [8]. Management strategies typically involve pre-surgical resuscitation, administration of broad-spectrum antibiotics, and urgent radiological assessment to confirm diagnosis [9]. Cecal volvulus necessitates swift intervention within 24-72 hours of diagnosis to mitigate mortality rates, which can exceed 30% [8]. Treatment options may include right hemicolectomy or ileocolic resection with ileostomy creation, followed by reversal once the patient's condition stabilizes [8]. Post-treatment complications, such as wound infection, sepsis, anastomotic leak, and prolonged ileus, contribute to elevated morbidity rates [8]. Gastric volvulus often presents with symptoms like nausea, vomiting, upper abdominal pain, and the classic Borchardt's triad [5]. Timely diagnosis and intervention are imperative to reduce morbidity and mortality rates [8]. While conservative measures like decompression and nasogastric tube insertion can be effective in some cases, surgical intervention is frequently warranted [8]. These clinical considerations underscore the critical importance of early detection, prompt treatment, and tailored management strategies across various types of volvulus to mitigate serious complications and enhance patient outcomes.

Clinical presentation

Common Signs and Symptoms

The signs and symptoms of volvulus, encompassing gastric and sigmoid types, exhibit variability contingent upon the specific condition. In the case of gastric volvulus, individuals may experience early satiety, dyspnea, chest discomfort, and dysphagia if the esophagogastric junction is impacted [10]. Acute gastric volvulus can manifest with Borchardt's triad, characterized by pain, retching, and the inability to pass a nasogastric tube, often accompanied by hiccups as a subtle indicator [10]. Conversely, chronic gastric volvulus may present as intermittent epigastric pain, abdominal fullness after meals, early satiety, and chest discomfort [10]. Conversely, sigmoid volvulus commonly exhibits symptoms such as abdominal distension, absolute constipation, and abdominal pain, with a sudden onset over a few hours and notable abdominal distension serving as prominent features [11]. Physical examination findings might reveal a distended abdomen, tympanic to percussion, along with signs indicative of peritonitis, suggestive of intestinal ischemia in advanced cases [11].

Atypical Presentations

Recognizing atypical presentations of illness, particularly in older adults, poses a significant challenge as they may deviate from the typical signs and symptoms associated with a specific condition. These atypical presentations often manifest as vague, altered, or absent symptoms. Notably, in older adults, changes in behavior or functional ability can serve as pivotal indicators of underlying serious illnesses, underscoring the importance of detecting these subtle changes early. Failure to identify atypical presentations can result in missed diagnoses, poorer outcomes, and lost opportunities to treat common conditions in this demographic [12,13]. The prevalence of atypical presentations escalates with age, underscoring the imperative for healthcare providers to maintain vigilance, particularly in individuals aged 85 years or older with multiple medical comorbidities and medications [12,13]. Recognizing atypical presentations necessitates a comprehensive evaluation, which includes careful observation of subtle changes in cognition and behavior. This task may be particularly challenging in patients with dementia, warranting input from reliable caregivers and family members [13]. Grasping and discerning atypical presentations of illness in older adults are pivotal to achieving timely diagnosis, implementing appropriate management strategies, and ultimately enhancing outcomes in this vulnerable population.

Importance of Early Recognition

The early recognition of gastric volvulus plays a pivotal role in ensuring favorable patient outcomes. This condition can present with nonbilious emesis, underscoring the critical importance of prompt identification to prevent potential complications. Early diagnosis enables timely intervention, mitigating the risk of gastric mucosal injury and ischemia. Incorporating endoscopy into the diagnostic process at an early stage allows for assessing ischemia in the gastric wall, facilitating the implementation of effective management strategies such as endoscopic reduction. Successful reduction of the volvulus via endoscopic procedures often results in a rapid resolution of symptoms. Surgical treatment may be warranted when gastric volvulus recurs frequently or is refractory to non-surgical interventions. Therefore, early recognition not only aids in a prompt diagnosis but also facilitates timely and appropriate interventions to manage gastric volvulus effectively [14,15]. This proactive approach minimizes the risk of complications and maximizes the chances of successful treatment outcomes, ultimately improving the overall prognosis for patients affected by this condition.

Diagnostic evaluation

Physical Examination Findings

Physical examination findings in patients with volvulus commonly include abdominal distention and tenderness. Patients often exhibit distress, accompanied by vital signs indicating tachycardia and a low-grade fever. Patients may present with stridor in gastric volvulus, where the stomach may be displaced into the chest cavity. Orthostatic hypotension and severe distension can progress to cardiogenic shock. Abdominal examination may reveal tympany, abdominal tenderness, rebound tenderness (positive Blumberg's sign), a palpable abdominal mass in various quadrants, and guarding. An empty left iliac fossa may suggest a sigmoid volvulus [16]. Examination of patients with sigmoid volvulus typically discloses significant abdominal distension, tympanitic percussion notes, and potential guarding over areas of ischemia or impending perforation. Signs of peritonitis may be present in cases where perforation has occurred [17]. These physical examination findings are crucial in guiding further diagnostic and management strategies for patients with volvulus.

Laboratory Investigations

Laboratory investigations play a pivotal role in both diagnosing and managing intestinal volvulus. A comprehensive approach to laboratory testing is indispensable when assessing patients suspected of obstruction. This typically encompasses a complete blood count, metabolic panel, and serum lactate level [18]. Specific findings in these tests can offer crucial insights into the patient's condition. For instance, hypokalemic hypochloremic metabolic alkalosis may indicate dehydration, while elevated blood urea nitrogen, hemoglobin, and hematocrit levels may further suggest dehydration [18]. Furthermore, the onset of metabolic acidosis, particularly when coupled with an increasing serum lactate level, can serve as a warning sign for bowel ischemia, necessitating urgent intervention [18]. In scenarios where concerns arise regarding abdominal sepsis, ischemia, or perforation, the identification of leukocytosis, leukopenia, and acidosis acts as red flags, mandating immediate surgical exploration [18]. When interpreted alongside clinical symptoms and imaging studies, these laboratory findings provide valuable guidance to the healthcare team in making timely and accurate decisions regarding the diagnosis and treatment of intestinal volvulus.

Radiological Modalities

Various radiological modalities are employed to evaluate abdominal volvulus, encompassing plain radiography, CT, USG, upper GI series, and nuclear imaging. Typically, plain radiography serves as the initial investigation, followed by CT scans, regarded as the gold standard for confirming the diagnosis of colonic volvulus [19]. In cases of gastric volvulus, USG emerges as a noninvasive and repeatable modality conducive to diagnosis, particularly in debilitated patients, although upper GI series are advocated for definitive confirmation [20]. Conversely, for sigmoid volvulus, radiography, fluoroscopy, and CT scans are commonly employed imaging methods for diagnosing and assessing the condition [21]. CT scans are pivotal in pinpointing specific findings, such as stenosis at the hernia neck and perigastric fluid in instances of gastric volvulus, thereby facilitating an accurate diagnosis and treatment planning [20]. Additionally, upper GI barium studies exhibit high sensitivity and specificity in diagnosing volvulus, furnishing valuable insights into the location and extent of the lesions [20]. These radiological modalities collectively contribute to a comprehensive diagnostic approach, aiding clinicians in effectively managing abdominal volvulus.

Role of Diagnostic Laparoscopy

The significance of diagnostic laparoscopy in evaluating and diagnosing various abdominal conditions must be balanced, particularly when traditional imaging studies fail to provide a definitive diagnosis. Laparoscopy is a minimally invasive approach to exploring the abdominal cavity, affording direct visualization of organs and tissues. This technique facilitates the identification of specific pathologies, enables the collection of tissue samples for biopsy, and allows for therapeutic interventions in select cases [22,23]. Diagnostic laparoscopy is invaluable in chronic abdominal conditions characterized by uncertain diagnoses, offering a safe and effective means to achieve conclusive diagnostic clarity with minimal complications and reduced operative time [22]. This procedure emerges as instrumental in scenarios where noninvasive diagnostic modalities present limitations or when direct visualization is warranted to confirm a diagnosis or assess the extent of a condition [24]. Overall, diagnostic laparoscopy is a versatile tool in the armamentarium of clinicians, providing a crucial avenue for elucidating complex abdominal pathologies and guiding appropriate therapeutic interventions.

Management principles

Initial Stabilization and Resuscitation

**Figure 2:**
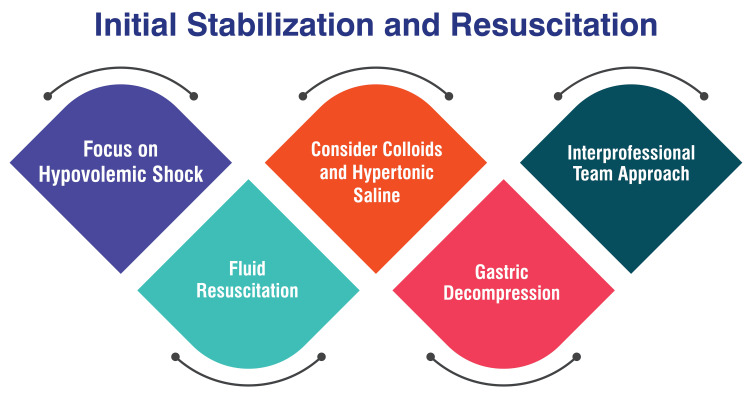
Initial stabilization and resuscitation The image is created by the corresponding author

During the initial stabilization phase, paramount attention is directed toward addressing hypovolemic shock, a critical concern stemming from decreased oxygen delivery to tissues. The therapeutic approach commences with administering oxygen therapy and establishing venous access to facilitate fluid resuscitation [25]. Fluid resuscitation utilizes isotonic crystalloid solutions such as lactate Ringer's solution (LRS), Normosol-R, or saline. Calculating and administering fluids at a shock rate (90 ml/kg/hr) is imperative, with continual reassessment after each bolus. The ultimate goal of fluid therapy is normalizing vital signs [25]. Considerations may be given to colloids and hypertonic saline in scenarios necessitating rapid or small-volume resuscitation. Colloids such as hetastarch can be administered at a dosage of 10-20 ml/kg. Alternatively, synthetic colloid and hypertonic saline may be employed for expedited resuscitation [25]. Following the initiation of volume resuscitation, attention should be directed toward gastric decompression. This may involve a combination of trocarization and orogastric intubation. While trocarization offers a swift and straightforward approach, it is full of risks. Conversely, orogastric intubation allows for complete decompression but carries the risk of aspiration pneumonia [25]. Effective management of gastric dilatation-volvulus (GDV) necessitates an interprofessional team comprising emergency physicians, surgeons, radiologists, and nurses. This collaborative approach ensures coordinated care delivery and optimizes outcomes for the patient [26]. Figure 2 shows initial stabilization and resuscitation.

Surgical Versus Non-surgical Management

Surgical intervention is the definitive treatment for colonic volvulus, encompassing procedures such as sigmoid resection with primary anastomosis or Hartmann's procedure [27]. Emergency surgery is warranted in cases of peritonitis or ischemic bowel, with the choice of procedure contingent upon intraoperative findings [27]. Specifically, for cecal volvulus, right hemicolectomy emerges as the preferred surgical approach, particularly in severely debilitated patients [27]. Studies have demonstrated that surgical management exhibits low mortality rates and effectively prevents the recurrence of volvulus, particularly in patients at a high risk of complications [28,29]. Non-operative management may be considered for acute sigmoid volvulus to mitigate surgical morbidity, especially in high-risk elderly and frail patients with unprepared bowels. However, this approach is associated with elevated recurrence and mortality rates [30]. Endoscopic therapy presents an effective alternative to surgery in appropriately selected patients devoid of colonic ischemia or perforations associated with colonic volvulus [30]. Primarily, conservative treatment should be employed to transition patients from an emergency surgery status to semi-elective surgery due to the elevated recurrence rates and mortality linked with non-operative management [30]. Notably, the endoscopic intervention has demonstrated lower inpatient mortality rates than surgical resection for colonic volvulus hospitalizations in the United States, possibly attributable to the invasive nature of the surgery and ensuing post-surgical complications [31]. Surgical management is frequently advocated for averting recurrence and diminishing morbidity associated with subsequent episodes of volvulus, especially in high-risk patients [28].

Timing of the Intervention

The timing of the intervention in cases of sigmoid volvulus emerges as a critical determinant significantly influencing patient outcomes. Extensive research underscores the pivotal role of prompt surgical intervention in reducing mortality rates associated with various clinical presentations of sigmoid volvulus. According to findings from a retrospective clinical study, patients exhibiting clear clinical signs of obstruction experienced a mortality rate of 44%, whereas those presenting with subocclusive symptoms faced a mortality rate of 35% [32]. The study further illuminated that mortality rates soared to 50% among the late-diagnosed patients who underwent surgery. Conversely, individuals treated with intestinal derotation and colpopexy exhibited no mortality [32]. Moreover, the prognosis of patients afflicted by sigmoid volvulus is intricately intertwined with the disease stage, the functional status of the patient, and their collaboration with clinicians in the pre-operative decision-making process. The study underscored that mortality rates tend to be higher in obstructed patients with generalized peritonitis and those affected by subocclusion with delayed diagnoses. In such dire circumstances, Hartmann's procedure often emerges as the recommended operation to consider [32]. This emphasizes the critical importance of timely surgical intervention in mitigating mortality rates and improving outcomes for patients grappling with sigmoid volvulus.

Patient-Specific Considerations

**Figure 3:**
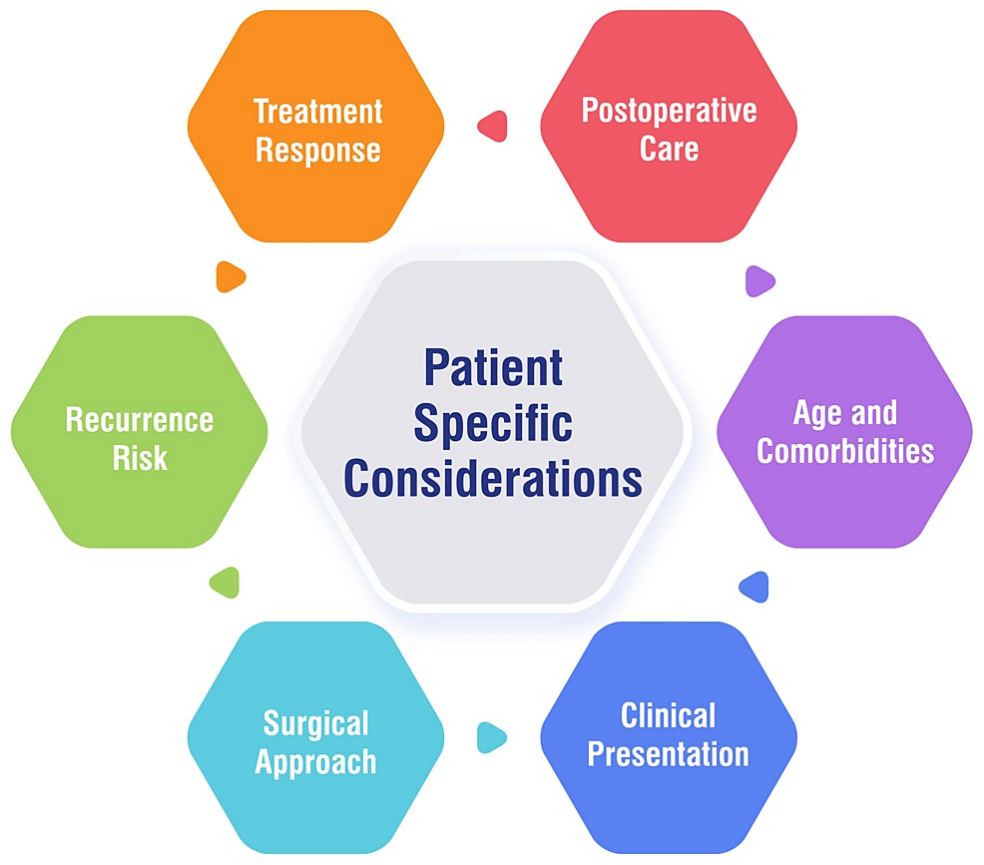
Patient-specific considerations The image is created by the corresponding author

Several factors play a significant role in managing volvulus, including the patient's age and comorbidities. Elderly individuals, particularly those over 60 years old, are at higher risk for volvulus, with age serving as a notable risk factor for mortality. Patients with neurological disorders, myopathies, or a history of previous volvulus episodes necessitate special attention and vigilant monitoring [33]. The clinical presentation of volvulus can vary widely, ranging from asymptomatic cases to severe peritonitis due to colonic perforation. The severity of symptoms and shock upon admission critically influence treatment decisions and subsequent outcomes [27,33]. Tailoring the surgical approach to each patient's clinical condition is paramount. Minimally invasive surgery may offer significant advantages, especially for elderly and chronically ill patients. However, the choice of surgical procedure ultimately hinges on individual patient factors and the severity of their condition [27]. Certain patient factors, such as chronic constipation, a high-fiber diet, neuropsychiatric disorders, and myopathies, can predispose individuals to volvulus recurrence. Patient education regarding these risk factors and preventive measures is essential for long-term management and reducing the likelihood of recurrence [4]. The response to initial treatment measures, such as endoscopic detorsion and decompression, can vary among patients. In cases where endoscopic reduction is contraindicated due to complications such as bowel gangrene or perforation peritonitis, immediate resuscitation, and surgical intervention are recommended to prevent further deterioration [4,27]. Effective postoperative care is crucial for optimal recovery. Educating patients and their relatives about colostomy and ileostomy care is paramount. Patient education on stoma care significantly contributes to recovery and helps reduce postoperative complications [27]. Figure 3 shows patient-specific considerations.

Non-operative management strategies

Detorsion Techniques

Detorsion techniques for sigmoid volvulus encompass non-operative approaches, notably endoscopic detorsion, which is the primary treatment for patients lacking peritonitis or perforation [34,35]. This technique, executed via flexible sigmoidoscopy, aims to gently navigate the endoscope through the twisted segment, facilitating aggressive decompression of the dilated colon and often resulting in spontaneous detorsion [35]. Endoscopic detorsion boasts a success rate ranging from 55% to 94%, proving particularly effective in pediatric patients [34,35]. Furthermore, placing a decompression tube proximal to the point of torsion post-detorsion is recommended to sustain reduction and promote continued colonic decompression [35]. In instances where endoscopic detorsion proves ineffective or in the presence of adverse events, immediate surgical intervention is warranted [35]. Overall, endoscopic detorsion emerges as a valuable technique in managing sigmoid volvulus, offering a non-operative option with a high success rate when performed under appropriate clinical conditions.

Decompression Methods

Various decompression methods are utilized to manage sigmoid volvulus, all aimed at alleviating obstruction and reducing the volvulus. Standard techniques include endoscopic detorsion, reduction, and tube decompression [36]. Endoscopic detorsion entails the use of a colonoscope to untwist the sigmoid colon, while tube decompression aids in relieving the obstruction and reducing the volvulus [36]. Furthermore, bedside decompression with a flatus tube should only be undertaken by experienced physicians under direct vision with rigid or flexible sigmoidoscopy to mitigate complications such as iatrogenic perforation [17]. Successful decompression typically results in the evacuation of liquid and gas per rectum, leading to resolution and a soft abdomen, which can be confirmed through repeat abdominal radiographs [17]. These decompression methods assume a pivotal role in managing sigmoid volvulus, particularly in stabilizing patients before contemplating surgical interventions. Their timely and effective implementation can significantly contribute to patient care and outcomes in cases of sigmoid volvulus.

Pharmacological Interventions

Pharmacological interventions are not the primary focus of managing acute sigmoid volvulus. For uncomplicated cases, the preferred treatment is non-operative detorsion, typically achieved through colonoscopic decompression or derotation [37]. However, if non-operative treatment fails or if complications such as peritonitis, gangrene, or perforation are present, emergency surgery becomes necessary [37]. Although pharmacological interventions do not form the cornerstone of treatment for sigmoid volvulus, broad-spectrum antibiotics with anaerobic coverage are administered to patients displaying signs of peritonitis, ischemic bowel, or sepsis as part of initial resuscitation measures [27]. These antibiotics aim to address potential infections and reduce the risk of complications associated with the condition. Overall, managing acute sigmoid volvulus predominantly revolves around mechanical interventions such as endoscopic decompression and surgical procedures, with pharmacological interventions supporting specific clinical scenarios.

Surgical management strategies

Options for the Surgical Approach (Open Vs. Laparoscopic)

The surgical management of acute gastric volvulus has demonstrated promising outcomes with laparoscopic repair, yielding improved postoperative clinical results and enhanced quality of life compared to open surgery [38]. Laparoscopy is considered a safe and efficient approach for treating acute gastric volvulus, with patients undergoing laparoscopic treatment reporting slightly superior quality of life scores compared to those undergoing open surgery [38]. Similarly, for acute sigmoid volvulus, non-operative detorsion is advocated as the primary treatment choice in uncomplicated cases, with colonoscopic derotation exhibiting a high success rate of 92.9% compared to other non-operative methods [37]. Conversely, in cases of cecal volvulus, emergency surgical intervention is often imperative, with right hemicolectomy emerging as the preferred surgical procedure, particularly in debilitated patients. This underscores the critical importance of prompt surgical management in such instances [27]. The decision between open and laparoscopic approaches for the surgical management of abdominal volvulus hinges on the specific type of volvulus, the condition of the patient, and the clinical presentation. Laparoscopic repair has demonstrated favorable outcomes for acute gastric volvulus, while non-operative colonoscopic derotation proves successful in numerous cases of acute sigmoid volvulus. Emergency surgical intervention, such as right hemicolectomy, is frequently necessary for cecal volvulus, emphasizing a tailored surgical approach based on the individual patient factors and the type of volvulus.

Specific Surgical Techniques for Different Types of Volvulus

Specific surgical techniques for different types of volvulus entail distinct approaches tailored to the particular condition. For cecal volvulus, cecopexy is a common procedure involving repositioning the cecum and its attachment to the abdominal wall. However, if the cecum sustains severe damage, intestinal resection surgery may be necessary [39]. Surgical options encompass sigmoid resection with primary anastomosis and the Hartmann's procedure in cases of sigmoid volvulus. The selection between these techniques is guided by intraoperative findings and the stability of the patient [40]. Endoscopic decompression serves as an initial step in managing sigmoid volvulus, with surgical intervention recommended for instances of peritonitis or ischemic bowel [40]. The management of cecal volvulus may necessitate a right hemicolectomy, particularly in debilitated patients. Cecostomy may be considered an alternative in critically ill patients [4]. Overall, the surgical management of volvulus demands tailored approaches based on the specific type of volvulus, the condition of the patient, and intraoperative findings to ensure optimal outcomes.

Considerations for Bowel Viability Assessment and Resection

In GI surgery, assessing bowel viability and determining resection is paramount to achieving optimal outcomes and minimizing postoperative complications. Various methods are available for the intraoperative assessment of intestinal viability, aiming to accurately delineate the extent of viable bowel to guide surgical resection. Crucial factors include the necessity for objective and quantitative techniques that are reproducible, cost-effective, and readily accessible in operating theaters dealing with abdominal emergencies [41,42]. When evaluating bowel viability, precision is essential, with minimal occurrence of false-negative and false-positive results. False-negative assessments may result in retaining nonviable bowel, risking complications such as perforation, while false-positive outcomes may lead to unnecessary resection of potentially salvageable intestine. Therefore, the chosen method must be objective, reproducible, and cost-effective to ensure precise decision-making during surgery [42]. Two widely acknowledged tests for assessing bowel viability are fluorescein assessment and Doppler studies, employing either ultrasound or laser velocimetry. These methods have demonstrated clinical applicability and acceptance due to their accuracy, reproducibility, and cost-effectiveness. Although other techniques may hold merit, the pragmatic approach often involves utilizing these established methods to assess bowel viability during surgery [42].

Postoperative care and complications

Monitoring and Management of Postoperative Complications

Following surgical procedures, monitoring, and managing postoperative complications constitute critical components of patient care. These complications, from cardiovascular and respiratory issues to infections and wound healing problems, necessitate diligent monitoring and prompt intervention to ensure optimal outcomes. Regular assessment of vital signs, pain levels, wound healing progress, mobility, diet, fluid balance, and bowel function is imperative during the early postoperative period [43]. Moreover, wearable devices can play a significant role in postoperative monitoring by continuously providing data on vital signs and other parameters. This capability enables healthcare teams to detect complications early and intervene promptly [43]. Guidelines underscore the importance of effectively stratifying and managing postoperative complications to minimize adverse outcomes and enhance patient recovery [44]. The cornerstone of postoperative care lies in close monitoring, selective interventions, and tailored management strategies to the specific complications encountered [45]. By implementing comprehensive monitoring protocols and leveraging wearable technology, healthcare providers can elevate postoperative care standards, identify complications early, and proactively intervene to optimize patient outcomes.

Long-Term Follow-up Strategies

Long-term follow-up strategies following surgeries for conditions such as volvulus are pivotal for monitoring patient outcomes and addressing potential complications. Studies underscore the significance of postoperative care, particularly in monitoring for complications like surgical site infections, anastomotic leakage, and sepsis, especially among older patients with volvulus [33]. Long-term follow-up should encompass assessing the patient's clinical condition, vigilant monitoring for signs of recurrence, and ensuring appropriate management of any postoperative complications that may emerge [33]. Moreover, for conditions like sigmoid volvulus, where endoscopic decompression followed by surgery are common approaches, long-term follow-up should prioritize evaluating the success of the initial treatment, determining the necessity for further interventions, and assessing the patient's quality of life post-surgery [33]. By adhering to comprehensive long-term follow-up protocols, healthcare providers can effectively track patient progress, identify potential issues early, and optimize management strategies to enhance patient outcomes and quality of life.

Recent advances and future directions

Emerging Technologies in Diagnosis and Treatment

Emerging technologies are revolutionizing healthcare practices, particularly in diagnosing and treating various medical conditions, including cancer and COVID-19. These innovations encompass diverse advancements, ranging from artificial intelligence (AI), machine learning, and deep learning to nanomedicine, 3D printing, and smart devices. Their integration is pivotal in enhancing diagnostic accuracy, tailoring personalized treatment approaches, and improving patient outcomes [46-50]. In cancer diagnostics, novel technologies like liquid biopsies, digital biopsies, and nanoparticle-based drug delivery systems are reshaping traditional management approaches. Liquid biopsies, for instance, offer non-invasive methods for disease monitoring by analyzing circulating tumor cells and DNA, thereby providing valuable insights for early detection and treatment monitoring [51]. Similarly, smart technologies such as smart contact lenses for glucose monitoring, smart toilets for health monitoring, and smartwatches for early detection of conditions like Parkinson's disease are transforming the landscape of disease diagnosis and management [49]. Furthermore, in the context of COVID-19, emerging technologies have played a pivotal role in patient study, diagnosis, and treatment. Collaborative efforts between medical researchers and engineers have led to rapid advancements in diagnostic tools, treatment strategies, and healthcare support systems. These developments underscore the critical importance of open access to knowledge and technology for mounting a timely response to global health crises [46].

Potential Areas for Further Research and Improvement

**Figure 4:**
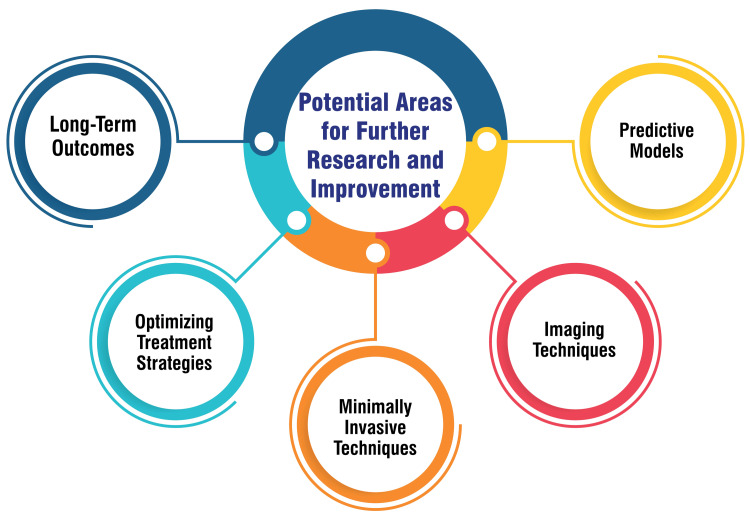
Potential areas for further research and improvement The image is created by the corresponding author

Further research can concentrate on refining and validating the predictive models such as the Malatya Volvulus Gangrene Model (MVGM) to enhance diagnostic accuracy and predict outcomes like bowel gangrene in sigmoid volvulus patients [33]. Continued advancements in imaging techniques, exemplified by the AXIS classification, can be explored to improve the assessment of the severity of sigmoid volvulus, thus enabling early intervention and potentially reducing morbidity and mortality rates [3]. Research into minimally invasive techniques for detorsion and decompression, including investigating the safety and efficacy of laparoscopic approaches in managing sigmoid volvulus, could improve outcomes and reduce patient complications [3,52]. Further studies can delve into optimal treatment strategies based on patient presentation, considering factors such as age, comorbidities, and clinical severity to tailor interventions effectively [52,53]. Research focusing on long-term outcomes post-treatment, recurrence rates, quality of life assessments, and factors influencing patient prognosis can provide valuable insights for enhancing overall care and management strategies [52,53]. Figure 4 shows potential areas for further research and improvement.

## Conclusions

In conclusion, this review has delved comprehensively into the management strategies for abdominal volvulus, highlighting the key aspects essential for clinical practice. By defining abdominal volvulus and emphasizing its clinical significance, we underscored the urgency of prompt recognition and appropriate intervention. Our exploration of diagnostic approaches and management options, including non-operative and surgical interventions, provides clinicians with a well-rounded understanding to effectively guide patient care. Furthermore, we emphasized the importance of a multidisciplinary approach in optimizing outcomes for patients with abdominal volvulus. Despite significant progress, unresolved issues persist, warranting further research. Refining diagnostic algorithms, enhancing surgical techniques, and exploring emerging technologies represent promising avenues for future investigation. By addressing these challenges and embracing future directions, we can advance the field and ultimately improve outcomes for individuals affected by abdominal volvulus.
